# Oxidative Stress Induced by Diclofenac Alone and under the Influence of Certain Variables in Broilers

**DOI:** 10.4103/0971-6580.68346

**Published:** 2010

**Authors:** C. Haritha, Reddy A. Gopala, Y. Anjaneyulu, B Kalakumar, Reddy G. Dilip

**Affiliations:** Department of Pharmacology and Toxicology, College of Veterinary Science, Rajendranagar, Hyderabad - 500 030, India; 1 Department of Veterinary Pathology, College of Veterinary Science, Rajendranagar, Hyderabad - 500 030, India

**Keywords:** Diclofenac, hepatotoxicity, high-protein high-calcium diet, oxidative stress

## Abstract

Diclofenac toxicity was studied in male broiler chicks (Cobb strain) of a day old age. The chicks were randomly divided into eight groups consisting of 10 in each group. Group 1 was kept as basal diet control (1–32 days), group 2 on basal diet for 32 days + diclofenac (0.8 mg/kg body weight I/M) on day 24, 26, 28, 30, and 32, group 3 on basal diet for 32 days + cyclophosphamide (50 mg/kg body weight I/M once daily) from day 20 to 23, group 4 on high-protein, high-calcium, low vitamin A (HPHC) diet (1–32 days), group 5 on basal diet + diclofenac + cyclophosphamide (as per the schedule), group 6 on HPHC + diclofenac (as per the schedule), group 7 on HPHC + cyclophosphamide (as per the schedule), and group 8 on HPHC + diclofenac + cyclophosphamide (as per the schedule). The activity of TBARS, SOD, and catalase revealed a significant (*P*< 0.05) increase, while there was a significant (*P* < 0.05) decrease in the concentration of GSH in liver in the groups given diclofenac either alone or in combination with other variables. There was no significant difference in the diclofenac residue levels. The electron microscopy of liver revealed lesions of mild to marked severity in different combinations. It can be concluded that diclofenac has the toxic potential in poultry at subtherapeutic doses and further the toxic effects were more pronounced under the influence of immunosuppressants and HPHC diet.

## INTRODUCTION

Diclofenac was a widely used veterinary drug (now licenses have been withdrawn) in the Indian subcontinent for the management of inflammation, fever, and/or pain associated with disease or injury in livestock. Vultures were exposed to diclofenac when scavenging on livestock treated with drug shortly before death[1] and lead to rapid decline by 97% in three species of vulture population namely *Gyps bengalensis, Gyps tenuirostris,* and *Gyps indicus*.[2] The proximate cause of decline was identified as being due to high rates of mortality from renal failure[3] and renal failure was found to be due to the toxic effect of diclofenac residues in livestock carcasses.[4] The first veterinary diclofenac product was registered in 1998 and vulture mortality due to visceral gout was already evident in 2000. Also bursal lymphoid depletion and epithelialization were found in five vultures and epithelial cysts were present in bursa of Fabricius of four vultures. However, they could not ascertain that the bursal regression was the only cause for immune suppression. Keeping all these reports in view, a comprehensive study was planned to investigate the toxicity, if any, due to diclofenac at subtherapeutic dose either alone or under the influence of certain variables like modified diet, i.e., high in protein, high in calcium, and low in vitamin A, and induced immunosuppression in broilers.

## MATERIALS AND METHODS

A total of 80 sexed male broiler chicks (Cobb strain) of a day old age were procured for the study. The chicks were randomly divided into eight groups consisting of 10 in each group. All the birds were provided with feed and water *ad libitum* throughout the experiment. All the groups were maintained as per the following treatment schedule for 5 weeks:

- Group 1. Basal diet control (1–32 days);
- Group 2. Basal diet for 32 days + diclofenac (0.8 mg/kg body weight I/M on day 24, 26, 28, 30, 32);
- Group 3. Basal diet for 32 days + cyclophosphamide (50 mg/kg body weight I/M once daily from day 20 to 23);
- Group 4. High-protein, high-calcium, low vitamin A diet control (HPHC) (1–32 days);
- Group 5. Basal diet + diclofenac + cyclophosphamide (as per the schedule);
- Group 6. High-protein, high-calcium, low vitamin A diet + diclofenac (as per the schedule);
- Group 7. High-protein, high-calcium, low vitamin A+ cyclophosphamide (as per the schedule);
- Group 8. High-protein, high-calcium, low vitamin A diet + diclofenac + cyclophosphamide (as per the schedule).

The basal diet contained 37% protein and 1% calcium whereas high-protein high-calcium low vitamin A (HPHC) diet contains 48% protein and 3% calcium. Both the diets were balanced energetically. Diclofenac was administered in groups 5 and 8 after confirming immunosuppression by performing HI and PHA tests. TBARS,[5] catalase,[6] glulathione (GSH),[7] and SOD[8] were estimated in liver tissue at the end of 34th day. Liver samples were collected for electron microscopy and diclofenac residue estimation.[9] Serum samples (*n* = 6) were separated for the estimation of protein profile, and renal and hepatic biomarkers by using commercially available diagnostic kits (Qualigens Pvt. Ltd., Mumbai). The data were analyzed by one-way ANOVA using statistical package for social sciences (SPSS) 10th version; *P*<0.05 was considered as significant.

## RESULTS AND DISCUSSION

The TBARS activity (nmoles MDA/g protein) of liver in the basal diet control (group 1) was 69.68±10.863, which was significantly (*P* < 0.05) increased in diclofenac-treated groups 2, 5, 6, and 8 [Table 1]. The TBARS activity in groups 3, 4, and 7 did not differ significantly as compared to basal diet control. The concentration of glutathione (GSH) (mg/g protein) of liver in the basal diet control (group 1) was 14.922±0.545, which was decreased significantly (*P* < 0.05) in diclofenac-treated groups 2, 5, 6, and 8. The GSH concentration was decreased significantly (*P* < 0.05) in group 3 (11.897 ± 0.629) as compared to basal diet control. The concentration of GSH in groups 4 and 7 (16.935 ± 1.935 and 13.490 ± 1.429, respectively) did not differ significantly (*P*<0.05) from the basal diet control. The SOD activity (units/g protein) of liver in the basal diet control was 42.365±3.113, which was significantly (*P* < 0.05) increased in diclofenac-treated groups 2, 5, 6, and 8. The SOD activity in groups 3 and 7 (52.647 ± 2.080 and 54.163 ± 1.598, respectively) was increased significantly (*P*<0.05), whereas group 4 (44.798 + 3.052) did not differ significantly as compared to that of basal diet control. The catalase activity (*μ*M/min) of liver in the basal diet control (group 1) was 2.197±0.319, which was significantly (*P* < 0.05) increased in diclofenac-treated groups 2, 5, 6, and 8. The catalase activity in groups 3 and 7 was increased significantly (*P*<0.05) as compared to basal diet control, whereas the activity of group 4 (1.862 ± 0.244) did not differ significantly as compared to group 1.

**Table 1:** Results of antioxidant parameters

Groups	TBARS activity (nM/g protein)	SOD activity (units/g protein)	Catalase activity (*μ*M/min)	GSH concentration (mg/g protein)
BASAL DIET	69.680±10.863^a^	42.365±3.113^a^	2.197±0.319^a^	14.922±0.545^e,f^
DCF	105.102±7.922^b^	58.273±2.134^b^	4.007±0.155^c^	10.235±0.438^b,c^
CP	62.749±3.554^a^	52.647±2.080^b^	2.832±0.230^b^	11.897±0.629^c,d^
HPHC DIET	65.731±4.609^a^	44.798±3.052^a^	1.862±0.244^a^	16.935±1.935^f^
DCF + CP	101.969±5.151^b^	73.168±2.152^c^	4.000±0.137^c^	5.791±0.654^a^
DCF + HPHC	91.786±7.366^b^	76.352±1.694^c^	4.708±0.207^d^	8.838±0.492^b^
HPHC + CP	67.697±5.380^a^	54.163±1.598^b^	2.995±0.222^b^	13.490±1.429^d,e^
DCF + HPHC + CP	111.957±7.000^b^	78.852±1.743^c^	4.822±0.178^d^	7.769±0.493^a,b^

Means with different superscripts are significantly different (*P*<0.05); DCF: diclofenac; CP: cyclophosphamide; Values are mean ± SE (*n* = 6); one way ANOVA (SPSS)

The significant increase in activity of TBARS, SOD, and catalase coupled with reduced levels of GSH in liver in diclofenac-treated groups indicates ongoing peroxidative stress and compromised antioxidant defense mechanisms. Hickey *et al*.[10] reported that diclofenac has the ability to provoke massive oxidative stress *in vivo*. The mechanism of diclofenac-induced mitochondrial injury seems to involve generation of ROS, causing oxidative stress to hepatocytes as proposed by Sokol *et al*.,[11] and number of structurally related NSAIDs do produce oxidative stress and the underlying mechanisms have been suggested to be based on peroxidase-catalyzed production of NSAID radicals, which in turn can oxidize GSH and NAD(P)H. NSAID radicals can undergo redox cycling.

**Figure 1:**
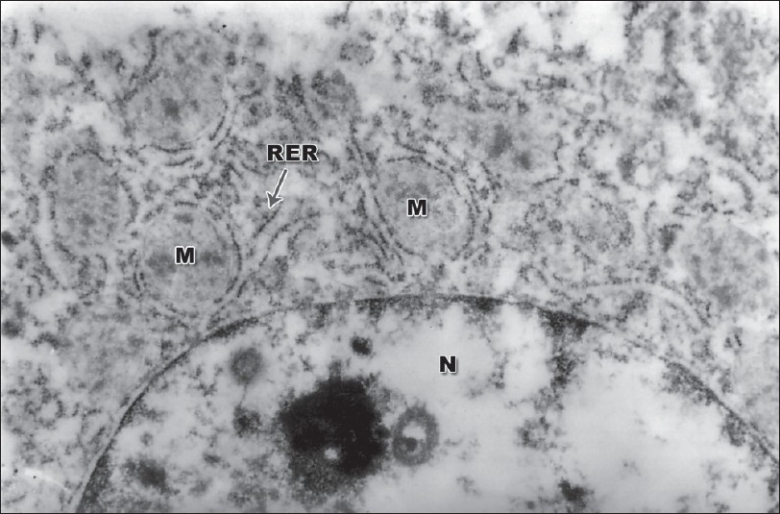
TEM of hepatocyte showing condensation of chromatin, bulged rough endoplasmic reticulum (RER), mitochondrial rupture (M). 26850×

**Figure 2:**
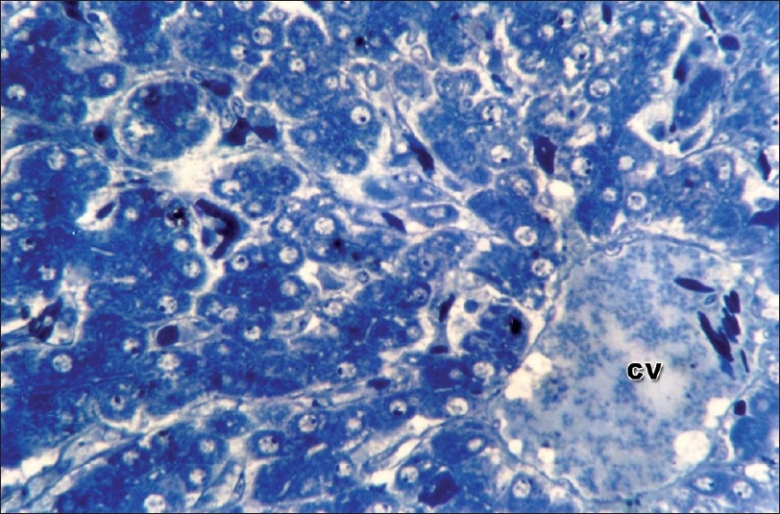
Semithin section of liver showing mild central vein congestion (CV), mild sinusoidal congestion. 630×

The concentration of diclofenac (*μ*g/g tissue) in liver of groups 2, 5, 6, and 8 was 0.031±0.012, 0.081 ± 0.002, 0.029 ± 0.0089, and 0.076 ± 0.033, respectively. In the groups that received diclofenac under the influence of cyclophosphamide (groups 5 and 6), there was nonsignificant elevation in the concentration of diclofenac residues in the liver suggesting poor degradation of the drug owing to ultrastructural damage to hepatocytes. These findings substantiated from the electron microscopy of hepatocyte of group 8, which revealed condensation of chromatin, bulged rough endoplasmic reticulum, and ruptured mitochondria [Figure 1]. Semithin sections of liver of group 8 showed central vein congestion, condensed hepatocytes, and reduced sinusoidal space [Figure 2]. The cytotoxicity of diclofenac appears to be associated with the CYP450 generation of a toxic metabolite that causes alteration in the mitochondrial function and depletion of ATP, thus inhibiting hepatic anabolic processes namely gluconeogenesis and protein synthesis.[12] Diclofenac has been reported to activate caspases 3, 8, and 9, which results in apotosis.[13] Diclofenac also uncouples oxidative phosphorylation and it is attributed to secondary amine in the structure.[14] Diclofenac is metabolized by CYP2C9 to hydroxylated metabolites, which may be further bioactivated to reactive iminoquinone intermediates, resulting in enhanced superoxide production and increased intracellular Ca ^2+^, which ultimately results in lethal cell injury.[15]

It can be concluded that diclofenac has the hepatotoxic potential in poultry at subtherapeutic doses, and further the toxic effects were more pronounced under the influence of immunosuppressants and high-protein high-calcium low vitamin A diet.
